# Supplementary material for: Content-rich biological network constructed by mining PubMed abstracts
Source: BMC Bioinformatics. 2004 Oct 8;5:147. doi: 10.1186/1471-2105-5-147 (PMC528731; doi:10.1186/1471-2105-5-147)
Supplement: Additional File 2 — The original results of the above study (non-essential files are deleted to keep the file size under the limit set by BMC bioinformatics). [file 1471-2105-5-147-S2.bz2 › chilibotAdditionalFile2/dip40/197ID8652557E680/html/ERCC3_GTF2H1.html]

 


 **ERCC3** and **GTF2H1** 
  
Found 427 abstracts in PubMed, retrieved 40.  
 

 What does Google say? 
 PDF only 
| .edu only 

---

**Interactive relationship** (e.g. stimulation, inhibition, etc)

**Neutral relationship**- Taken together, our results show that the p52 Tfb2 subunit of TFIIH  [ **GTF2H1** ]   [ **ERCC3** ]  regulates the function of XPB  [ **ERCC3** ]  through pair wise interactions as described previously for p44 and XPD.  Ref: 12080057 J Biol Chem, 2002
- We established that the carboxy terminal 125 amino acids are dispensable for both HR23B and damaged DNA binding, while interactions with transcription factor IIH TFIIH  [ **GTF2H1** ]   [ **ERCC3** ]  are significantly impaired by truncation of this domain.  Ref: 12509233 DNA Repair Amst , 2002
- Using computer analysis we identified several common motifs in the p62 proteins from different organisms, suggesting that these motifs could be involved in possible protein protein interactions within the TFIIH  [ **GTF2H1** ]   [ **ERCC3** ]  complex or with other transcription and DNA repair factors.  Ref: 12509240 DNA Repair Amst , 2002
- Trichothiodystrophy TTD is a rare hereditary multisystem disorder associated with defects in nucleotide excision repair NER as a consequence of mutations in XPD, XPB  [ **ERCC3** ]  or TTDA, three genes that are all related to TFIIH  [ **GTF2H1** ]   [ **ERCC3** ] , the multiprotein complex involved in NER and transcription.  Ref: 12393803 Hum Mol Genet, 2002
- The dual role of TFIIH  [ **GTF2H1** ]   [ **ERCC3** ]  in DNA repair and transcription makes it difficult to discern which of the mutant TFIIH  [ **GTF2H1** ]   [ **ERCC3** ]  phenotypes is due to defects in any of these different processes.  Ref: 12221129 Mol Biol Cell, 2002
- We also show that mutations in CSB, as well as in XPB  [ **ERCC3** ]  and XPD genes, all of which confer CS, disturb the RNA pol I TFIIH  [ **GTF2H1** ]   [ **ERCC3** ]  interaction within the CSB IP 150.  Ref: 12419226 Mol Cell, 2002
- Analyses of AR mediated transcription suggest that the orchestrated interaction of AR with the transcription factors IIF TFIIF and IIH TFIIH  [ **GTF2H1** ]   [ **ERCC3** ] , and positive transcription elongation factor b P TEFb, may increase efficiency of transcriptional elongation from the androgen target genes, such as prostate specific antigen PSA.  Ref: 12648523 J Steroid Biochem Mol Biol, 2003
- Recent advances have shed some light on the structure of these factors, how conformational changes and intramolecular interactions regulate activity, and have revealed an expanded role for TFIIH  [ **GTF2H1** ]   [ **ERCC3** ]  in nuclear transcription.  Ref: 12672487 Curr Opin Genet Dev, 2003
- Moreover, when XPD mutations prevent interaction with the p44 subunit of TFIIH  [ **GTF2H1** ]   [ **ERCC3** ] , transactivation directed by certain nuclear receptors is inhibited, regardless of TTD versus XP phenotype, thus explaining the overlapping symptoms.  Ref: 12820975 Mol Cell, 2003
- ... TFIIH  [ **GTF2H1** ]   [ **ERCC3** ]  overexpression did not potentiate the transcriptional activity of a Vpr mutant, which does not bind p300 CBP.  Ref: 12379213 Biochem Biophys Res Commun, 2002
- These findings suggest that TFIIH  [ **GTF2H1** ]   [ **ERCC3** ]  participates in Vpr s GR coactivating activity, at a step beyond its interaction with p300 CBP.  Ref: 12379213 Biochem Biophys Res Commun, 2002
- General transcription factor IIH TFIIH  [ **GTF2H1** ]   [ **ERCC3** ]  consists of nine subunits cyclin dependent kinase 7 Cdk7, cyclin H and MAT1 forming the Cdk activating kinase or CAK complex, the two helicases Xpb  [ **ERCC3** ]  Hay and Xpd, and p34, p44, p52 and p62 refs 1 3.  Ref: 12853965 Nature, 2003
- Thus, TFIIH  [ **GTF2H1** ]   [ **ERCC3** ]  appears to protect promoters from PC4 mediated repression by relieving the topological constraint imposed by PC4 through the  **ERCC3**  helicase activity rather than by reducing the repressive activity of PC4 via its phosphorylation.  Ref: 12590132 J Biol Chem, 2003

**Non-interactive relationship** (e.g. studied together, co-existance, homology, etc.)

- Together with recent reports linking TFIIH  [ **GTF2H1** ]   [ **ERCC3** ]  in regulating NR function, our findings provide new data to further support an important link between DNA repair proteins and nuclear receptor function.  Ref: 12874288 J Biol ChemJ Biol Chem, 2003
- To further our understanding of the transcription DNA repair factor TFIIH  [ **GTF2H1** ]   [ **ERCC3** ] , we investigated the role of its p52 subunit in TFIIH  [ **GTF2H1** ]   [ **ERCC3** ]  function.  Ref: 12080057 J Biol Chem, 2002
- The kinase subunits of TFIIH  [ **GTF2H1** ]   [ **ERCC3** ]  in animal cells, Cdk7, cyclin H, and MAT1, were independently isolated as an activity termed CAK Cdk activating kinase, which phosphorylates and activates cell cycle kinases.  Ref: 12176978 J Biol Chem, 2002
- Set1 association depends upon the TFIIH  [ **GTF2H1** ]   [ **ERCC3** ]  associated kinase that phosphorylates the Pol II C terminal domain CTD and mediates the transition between initiation and elongation, and Set1 interacts with the form of Pol II whose CTD is phosphorylated at serine 5 but not serine 2.  Ref: 12667453 Mol Cell, 2003
- Conditional ablation of the Mat1 subunit of TFIIH  [ **GTF2H1** ]   [ **ERCC3** ]  in Schwann cells provides evidence that Mat1 is not required for general transcription.  Ref: 12376559 J Cell Sci, 2002
- Analysis of the TFIIH  [ **GTF2H1** ]   [ **ERCC3** ]  dependent stages of transcription in a reconstituted system demonstrates that U1 stimulates the rate of formation of the first phosphodiester bond by RNA polymerase II.  Ref: 12389039 Nat Struct Biol, 2002
- Here we show that all the mutations found in TTD cases, irrespective of whether they are homozygotes, hemizygotes or compound heterozygotes, cause a substantial and specific reduction by up to 70% in the cellular concentration of TFIIH  [ **GTF2H1** ]   [ **ERCC3** ] .  Ref: 12393803 Hum Mol Genet, 2002
- Using recombinant TFIIH  [ **GTF2H1** ]   [ **ERCC3** ] , we have analyzed the role of TFIIH  [ **GTF2H1** ]   [ **ERCC3** ]  for alleviating PC4 mediated transcriptional repression and determined that the excision repair cross complementing  **ERCC3**  helicase activity of TFIIH  [ **GTF2H1** ]   [ **ERCC3** ]  is the enzymatic activity that alleviates PC4 mediated repression via beta gamma bond hydrolysis of ATP.  Ref: 12590132 J Biol Chem, 2003
- Intriguingly, the degree of reduction in the level of TFIIH  [ **GTF2H1** ]   [ **ERCC3** ]  does not correlate with the severity of the pathological phenotype, suggesting that the severity of the clinical features in TTD cannot be related solely to the effects of mutations on the stability of TFIIH  [ **GTF2H1** ]   [ **ERCC3** ] .  Ref: 12393803 Hum Mol Genet, 2002
- Mutations in these residues also affected the ability of hTFIIE beta to stimulate TFIIH  [ **GTF2H1** ]   [ **ERCC3** ]  mediated phosphorylation of the carboxy terminal heptapeptide repeats of the largest subunit of Pol II.  Ref: 12665589 Mol Cell Biol, 2003
- Moreover, we demonstrate that intact p52 is needed to anchor the XPB  [ **ERCC3** ]  helicase within TFIIH  [ **GTF2H1** ]   [ **ERCC3** ] , providing an explanation for the transcription and NER defects observed with the mutant p52.  Ref: 12080057 J Biol Chem, 2002
- The mutant Tfb3 protein associates less efficiently with the kinase moiety of TFIIH  [ **GTF2H1** ]   [ **ERCC3** ]  than the wild type protein.  Ref: 12176978 J Biol Chem, 2002
- Three polypeptides, primarily positioned 5 to the damage, are in close physical proximity to the psoralen lesion and thus are cross linked to the damaged DNA these proteins are RPA70, RPA32, and the XPD subunit of TFIIH  [ **GTF2H1** ]   [ **ERCC3** ] .  Ref: 12138203 Mol Cell Biol, 2002
- TFIIH  [ **GTF2H1** ]   [ **ERCC3** ]  was homogeneously distributed throughout the nucleus with nucleolar accumulations.  Ref: 12453423 Mol Cell, 2002
- Furthermore, these mutations conspicuously augmented the binding of hTFIIE beta to the p44 subunit of TFIIH  [ **GTF2H1** ]   [ **ERCC3** ] .  Ref: 12665589 Mol Cell Biol, 2003
- Rapid switching of TFIIH  [ **GTF2H1** ]   [ **ERCC3** ]  between RNA polymerase I and II transcription and DNA repair in vivo.  Ref: 12453423 Mol Cell, 2002
- Colocalization experiments of the NER factors showed that in normal human cells, XPC and TFIIH  [ **GTF2H1** ]   [ **ERCC3** ]  are rapidly and efficiently recruited to DNA damage within SNS.  Ref: 12713809 DNA Repair Amst , 2003
- This mutation prevents promoter opening and has no effect on the other enzymatic activities of TFIIH  [ **GTF2H1** ]   [ **ERCC3** ] .  Ref: 12080057 J Biol Chem, 2002
- Analysis of the results concentrated on the ring finger domain as the classic zinc finger domains were too constrained to provide much topological variety, whilst the TFIIH  [ **GTF2H1** ]   [ **ERCC3** ]  domain which has large unstructured loops did not behave well.  Ref: 12527307 J Mol Biol, 2003
- The antibody study indicated that they thus altered the conformation of one side of TFIIH  [ **GTF2H1** ]   [ **ERCC3** ] , consisting of p44, XPD, and Cdk activating kinase subunits, that is essential for the transition stage.  Ref: 12665589 Mol Cell Biol, 2003
- In addition, a promoter proximal 5 splice site recognized by U1 snRNA stimulates TFIIH  [ **GTF2H1** ]   [ **ERCC3** ]  dependent reinitiation of productive transcription.  Ref: 12389039 Nat Struct Biol, 2002
- We also show that TFIIH  [ **GTF2H1** ]   [ **ERCC3** ]  from TTD patients, but not from XP patients, exhibits a significant in vitro basal transcription defect in addition to a reduced intracellular concentration.  Ref: 12820975 Mol Cell, 2003
- A CDK7 mutant defective in kinase activity for the C terminal tail of RNA polymerase II, which cannot form a functional TFIIH  [ **GTF2H1** ]   [ **ERCC3** ]  complex, did not enhance Vpr coactivator activity.  Ref: 12379213 Biochem Biophys Res Commun, 2002
- Alleviation of PC4 mediated Transcriptional Repression by the  **ERCC3**  Helicase Activity of General Transcription Factor TFIIH  [ **GTF2H1** ]   [ **ERCC3** ] .  Ref: 12590132 J Biol Chem, 2003
- The spatial distribution of proteins involved in transcription TFIIH  [ **GTF2H1** ]   [ **ERCC3** ]  and RNA polymerase II and RNA processing and packaging hnRNP U were analyzed in relation to chromosome territories and large scale chromatin organization.  Ref: 12364563 J Histochem Cytochem, 2002
- U1 snRNA associates with TFIIH  [ **GTF2H1** ]   [ **ERCC3** ]  and regulates transcriptional initiation.  Ref: 12389039 Nat Struct Biol, 2002
- To study TFIIH  [ **GTF2H1** ]   [ **ERCC3** ]  in vivo, we generated cell lines expressing functional GFP tagged TFIIH  [ **GTF2H1** ]   [ **ERCC3** ] .  Ref: 12453423 Mol Cell, 2002
- Interestingly, some of the symptoms in afflicted individuals appear to be due to defects in transcription, the result of the dual functionality of several components of the NER apparatus as parts of transcription factor IIH TFIIH  [ **GTF2H1** ]   [ **ERCC3** ] .  Ref: 12488584 J Biomed Biotechnol< MedlineTA>J Biomed Biotechnol, 2002
- Together, AR may be able to interact with TFIIH  [ **GTF2H1** ]   [ **ERCC3** ] , P TEFb, and RPB2 to enhance transcription from AR target genes, such as prostate specific antigen that may play important roles in the prostate cancer progression.  Ref: 12593864 Biochem Biophys Res Commun, 2003
- Core NER enzymes XPA, XPF, XPG, and TFIIH  [ **GTF2H1** ]   [ **ERCC3** ]  are essential for recovery.  Ref: 12531012 DNA Repair Amst , 2002
- Here we show that the Drosophila TFIIH  [ **GTF2H1** ]   [ **ERCC3** ]  component Xpd negatively regulates the cell cycle function of Cdk7, the CAK activity.  Ref: 12853965 Nature, 2003
- Transcription factor TFIIH  [ **GTF2H1** ]   [ **ERCC3** ]  components enhance the GR coactivator activity but not the cell cycle arresting activity of the human immunodeficiency virus type 1 protein Vpr.  Ref: 12379213 Biochem Biophys Res Commun, 2002
- These include formation of abortive transcripts, strong dependence on ATP cofactor, the general transcription factor TFIIH  [ **GTF2H1** ]   [ **ERCC3** ]  and downstream template.  Ref: 12213653 Biochim Biophys Acta, 2002
- The preferred substrate for SCP1 is RNAP II phosphorylated by TFIIH  [ **GTF2H1** ]   [ **ERCC3** ] .  Ref: 12721286 J Biol Chem< MedlineTA>J Biol Chem, 2002
- We have previously reported that the susceptibility gene for DPB could be localized within a 200 kb segment between the S and TFIIH  [ **GTF2H1** ]   [ **ERCC3** ]  loci in the HLA class I region, using refined microsatellite based association mapping.  Ref: 12185533 Immunogenetics, 2002
- We have found mild reductions up to 40% in TFIIH  [ **GTF2H1** ]   [ **ERCC3** ]  content in some but not all of these cell strains.  Ref: 12393803 Hum Mol Genet, 2002
- We also show that, as complexed within TFIIH  [ **GTF2H1** ]   [ **ERCC3** ] , the cyclin dependent kinase 7 kinase does not possess the activity to phosphorylate PC4.  Ref: 12590132 J Biol Chem, 2003
- The mammalian Mat1 protein has been implicated in cell cycle regulation as part of the Cdk activating kinase CAK, and in regulation of transcription as a subunit of transcription factor TFIIH  [ **GTF2H1** ]   [ **ERCC3** ] .  Ref: 12376559 J Cell Sci, 2002
- The transcription repair factor TFIIH  [ **GTF2H1** ]   [ **ERCC3** ]  operates as a DNA helix opener in RNA polymerase II RNAP2 transcription and nucleotide excision repair.  Ref: 12453423 Mol Cell, 2002
- Mutations in XPB  [ **ERCC3** ]  and XPD TFIIH  [ **GTF2H1** ]   [ **ERCC3** ]  helicases have been related with three hereditary human disorders xeroderma pigmentosum, Cockayne syndrome, and trichothiodystrophy.  Ref: 12221129 Mol Biol Cell, 2002
- We conclude that the severity of the clinical features in TTD patients and the clinical outcome of differentially mutated XPD proteins is likely to depend both on the effects that each mutation has on the stability of TFIIH  [ **GTF2H1** ]   [ **ERCC3** ]  and on the transcriptional activity of the residual TFIIH  [ **GTF2H1** ]   [ **ERCC3** ]  complexes.  Ref: 12393803 Hum Mol Genet, 2002
- Cyclin dependent kinase 8 cdk8 regulates transcription by phosphorylating RNA polymerase II and TFIIH  [ **GTF2H1** ]   [ **ERCC3** ] .  Ref: 12031503 Biochim Biophys Acta, 2002
- Photobleaching revealed that TFIIH  [ **GTF2H1** ]   [ **ERCC3** ]  moves freely and gets engaged in RNAP1 and RNAP2 transcription for approximately 25 and approximately 6 s, respectively.  Ref: 12453423 Mol Cell, 2002
- Mutations in some components of TFIIH  [ **GTF2H1** ]   [ **ERCC3** ]  are associated with three hereditary human syndromes xeroderma pigmentosum XP, Cockayne syndrome CS and trichothiodystrophy TTD.  Ref: 12509240 DNA Repair Amst , 2002
- Human nucleotide excision repair is initiated by six repair factors XPA, RPA, XPC HR23B, TFIIH  [ **GTF2H1** ]   [ **ERCC3** ] , XPF ERCC1, and XPG which sequentially assemble at sites of DNA damage and effect excision of damage containing oligonucleotides.  Ref: 12138203 Mol Cell Biol, 2002
- We demonstrate that TFIIH  [ **GTF2H1** ]   [ **ERCC3** ] , a general transcription initiation factor, specifically associates with U1 snRNA, a core splicing component.  Ref: 12389039 Nat Struct Biol, 2002
- The results also showed that dispersal of recruited XPC and TFIIH  [ **GTF2H1** ]   [ **ERCC3** ]  from DNA damage SNS occurs within a short period after DNA damage.  Ref: 12713809 DNA Repair Amst , 2003
- Here, it is shown that the APIS complex is physically associated with many general transcription factors, including components of yeast FACT Cdc68 Pob3, TFIID, TFIIH  [ **GTF2H1** ]   [ **ERCC3** ] , and the RNA polymerase II holoenzyme.  Ref: 12200147 Biochem Biophys Res Commun, 2002
- By using a chromatin immunoprecipitation technique, we found that TFIIE is necessary for stable binding of TFIIH  [ **GTF2H1** ]   [ **ERCC3** ]  and RNAPII to the ADH1 promoter, whose activation requires TFIIE.  Ref: 12558798 Genes Cells, 2003
- Molecular characterization and developmental expression of the TFIIH  [ **GTF2H1** ]   [ **ERCC3** ]  factor p62 gene from Drosophila melanogaster effects on the UV light sensitivity of a p62 mutant fly.  Ref: 12509240 DNA Repair Amst , 2002
- Here, we provide evidence that CSB is found not only in the nucleoplasm but also in the nucleolus within a complex CSB IP 150 that contains RNA pol I, TFIIH  [ **GTF2H1** ]   [ **ERCC3** ] , and XPG and promotes efficient rRNA synthesis.  Ref: 12419226 Mol Cell, 2002
- Tumor suppressor p53 dependent recruitment of nucleotide excision repair factors XPC and TFIIH  [ **GTF2H1** ]   [ **ERCC3** ]  to DNA damage.  Ref: 12713809 DNA Repair Amst , 2003
- The presence of other repair factors, in particular TFIIH  [ **GTF2H1** ]   [ **ERCC3** ] , alters the mode of RPA binding and the position of its subunits relative to the psoralen lesion.  Ref: 12138203 Mol Cell Biol, 2002
- The p62 protein is a structural component of the TFIIH  [ **GTF2H1** ]   [ **ERCC3** ]  core and no syndromes have been identified up to date by mutations in this human gene.  Ref: 12509240 DNA Repair Amst , 2002
- In this study, the regulatory effect of p53, on the spaciotemporal recruitment of XPC and TFIIH  [ **GTF2H1** ]   [ **ERCC3** ]  to DNA damage sites, was investigated in repair proficient and deficient human cells in situ.  Ref: 12713809 DNA Repair Amst , 2003
- Taken together, the results demonstrated that p53 plays a pronounced role in the damage recognition and subsequent assembly of repair machinery during GGR and the recruitment of XPC and TFIIH  [ **GTF2H1** ]   [ **ERCC3** ]  to CPD is p53 dependent.  Ref: 12713809 DNA Repair Amst , 2003
- The archaeal transcriptional machinery is polII like but does not require ATP or TFIIH  [ **GTF2H1** ]   [ **ERCC3** ]  for open complex formation.  Ref: 12783891 J Biol Chem< MedlineTA>J Biol Chem, 2003
- We have recently completed screening of the National Cancer Institute human tumor cell line panel and demonstrated that among four nucleotide excision repair proteins XPA, XPB  [ **ERCC3** ] , XPD, and ERCC1, only the TFIIH  [ **GTF2H1** ]   [ **ERCC3** ]  subunit XPD endogenous protein levels correlate with alkylating agent drug resistance.  Ref: 12359753 Cancer Res, 2002
- Previous studies showed that the repressive activity of PC4 on promoter driven transcription is alleviated by transcription factor TFIIH  [ **GTF2H1** ]   [ **ERCC3** ] , possibly through one of its enzymatic activities.  Ref: 12590132 J Biol Chem, 2003
- Often, phosphorylation of NRs by kinases that are associated with general transcription factors e.g. cdk7 within TFIIH  [ **GTF2H1** ]   [ **ERCC3** ] , or activated in response to a variety of signals MAPKs, Akt, PKA, PKC, facilitates the recruitment of coactivators or of components of the transcription machinery and, therefore, cooperates with the ligand to enhance transcription activation.  Ref: 12618210 Cell Signal, 2003
- We provide in vivo evidence for involvement of TFIIH  [ **GTF2H1** ]   [ **ERCC3** ]  in RNA polymerase I RNAP1 transcription.  Ref: 12453423 Mol Cell, 2002
- Regulation of cisplatin resistance and homologous recombinational repair by the TFIIH  [ **GTF2H1** ]   [ **ERCC3** ]  subunit XPD.  Ref: 12359753 Cancer Res, 2002
- As the kinase subunit of TFIIH  [ **GTF2H1** ]   [ **ERCC3** ] , Cdk7 participates in basal transcription by phosphorylating the carboxy terminal domain of the largest subunit of RNA polymerase II.  Ref: 12853965 Nature, 2003
- Overexpression of all three TFIIH  [ **GTF2H1** ]   [ **ERCC3** ]  components and p300 cooperatively enhanced Vpr coactivator activity.  Ref: 12379213 Biochem Biophys Res Commun, 2002
- In this report, we demonstrate an association of a C terminal domain of human RAD52 amino acids 302 418 with the XPB  [ **ERCC3** ]  and XPD subunits of transcription factor TFIIH  [ **GTF2H1** ]   [ **ERCC3** ]  and RNA polymerase II RNAPII.  Ref: 12372413 Biochem Biophys Res Commun, 2002
- However, CAK activity of TFIIH  [ **GTF2H1** ]   [ **ERCC3** ]  subunits could not be demonstrated in budding yeast.  Ref: 12176978 J Biol Chem, 2002
- In contrast to lethal mutants in other subunits of TFIIH  [ **GTF2H1** ]   [ **ERCC3** ] , this mutation does not impair general transcription.  Ref: 12176978 J Biol Chem, 2002
- XPA and RPA, once forming a complex at the damage site, would likely work with TFIIH  [ **GTF2H1** ]   [ **ERCC3** ] , XPG, and ERCC1 XPF for dual incision.  Ref: 12486030 J Biol Chem, 2003
- TFIIH  [ **GTF2H1** ]   [ **ERCC3** ]  readily switches between transcription and repair sites where it is immobilized for approximately 4 min without large scale alterations in composition.  Ref: 12453423 Mol Cell, 2002
- DNA repair and transcriptional effects of mutations in TFIIH  [ **GTF2H1** ]   [ **ERCC3** ]  in Drosophila development.  Ref: 12221129 Mol Biol Cell, 2002
- To conclude, in the present study we bring another demonstration of the multifunctional nature of TFIIH  [ **GTF2H1** ]   [ **ERCC3** ] .  Ref: 12176978 J Biol Chem, 2002
- MAT1 is an assembly targeting factor for cyclin dependent kinase activating kinase CAK, which has been shown to functionally interact with general transcriptional factor TFIIH  [ **GTF2H1** ]   [ **ERCC3** ] , a known inducer of ER transactivation.  Ref: 12527756 J Biol Chem, 2003
- These results suggest that following initial damage recognition, the carboxy terminus of XPC may be essential for the recruitment of TFIIH  [ **GTF2H1** ]   [ **ERCC3** ] ,.  Ref: 12509233 DNA Repair Amst , 2002
- By contrast, recruitment of both XPC and TFIIH  [ **GTF2H1** ]   [ **ERCC3** ]  to DNA damage in SNS occurred much less efficiently in p53 Null or p53 compromised cells.  Ref: 12713809 DNA Repair Amst , 2003
- Using a completely reconstituted in vitro transcription or nucleotide excision repair NER system, we show that deletion of the C terminal region of p52 results in a dramatic reduction of TFIIH  [ **GTF2H1** ]   [ **ERCC3** ]  NER and transcription activities.  Ref: 12080057 J Biol Chem, 2002
- XPA, XPC hHR23B, RPA, and TFIIH  [ **GTF2H1** ]   [ **ERCC3** ]  all are the damage recognition proteins essential for the early stage of nucleotide excision repair.  Ref: 12486030 J Biol Chem, 2003
- However, binding of TFIIH  [ **GTF2H1** ]   [ **ERCC3** ]  and RNAPII to CUP1 upon its activation did not require TFIIE.  Ref: 12558798 Genes Cells, 2003
- Our previous reports demonstrated that AR interacted with TFIIH  [ **GTF2H1** ]   [ **ERCC3** ]  and positive transcription elongation factor b P TEFb,.  Ref: 12593864 Biochem Biophys Res Commun, 2003
- TFIIH  [ **GTF2H1** ]   [ **ERCC3** ]  is a multiprotein complex that has a central role in the RNA pol II mediated transcription, in DNA repair and in the control of the cell cycle.  Ref: 12509240 DNA Repair Amst , 2002
- We have also measured TFIIH  [ **GTF2H1** ]   [ **ERCC3** ]  levels in cells in which different mutations in the XPD gene are associated with clinical symptoms not of TTD but of the highly cancer prone disorder xeroderma pigmentosum XP.  Ref: 12393803 Hum Mol Genet, 2002
- TFB3, the 38 kDa subunit of yeast TFIIH  [ **GTF2H1** ]   [ **ERCC3** ] , is the homolog of mammalian MAT1.  Ref: 12176978 J Biol Chem, 2002
- p52 Mediates XPB  [ **ERCC3** ]  function within the transcription repair factor TFIIH  [ **GTF2H1** ]   [ **ERCC3** ] .  Ref: 12080057 J Biol Chem, 2002
- Reduced level of the repair transcription factor TFIIH  [ **GTF2H1** ]   [ **ERCC3** ]  in trichothiodystrophy.  Ref: 12393803 Hum Mol Genet, 2002
- To achieve CTD hyperphosphorylation, Tat stimulates CTD kinases associated with general transcription factors of the promoter complex, specifically TFIIH  [ **GTF2H1** ]   [ **ERCC3** ]  associated CDK7 and positive transcription factor b associated CDK9 cyclin dependent kinase 9.  Ref: 12049628 Biochem J, 2002
- The RNA polymerase II general transcription factor TFIIH  [ **GTF2H1** ]   [ **ERCC3** ]  is composed of 9 known subunits and possesses DNA helicase and protein kinase activities.  Ref: 12176978 J Biol Chem, 2002
- Expression of several nuclear genes involved in cell signaling, cell architecture, energy metabolism, cell growth, apoptosis including general transcription factor TFIIH  [ **GTF2H1** ]   [ **ERCC3** ] , v maf, AML1, was induced in Rho0 cells.  Ref: 12495447 Mol Cancer< MedlineTA>Mol Cancer, 2002
